# Prevalence and risk factors associated with *Eimeria* species infection in cattle of different geographical regions of Indonesia

**DOI:** 10.14202/vetworld.2021.2339-2345

**Published:** 2021-09-06

**Authors:** Fitrine Ekawasti, Raden Wisnu Nurcahyo, Lintang Winantya Firdausy, April Hari Wardhana, Dyah Haryuningtyas Sawitri, Joko Prastowo, Dwi Priyowidodo

**Affiliations:** 1 Department of Parasitology, Faculty of Veterinary Medicine, Universitas Gadjah Mada, Yogyakarta, 55281, Indonesia;; 2 Indonesian Research Center for Veterinary Sciences, Indonesian Agency for Agricultural Research and Development, Ministry of Agriculture Republic Indonesia, Bogor, 16167, Indonesia.

**Keywords:** cattle, *Eimeria*, floatation, gastrointestinal, risk factor

## Abstract

**Background and Aim:**

*Eimeria* spp. are gastrointestinal protozoans that affect animal productivity, thereby causing symptoms that range from bloody diarrhea to death. These symptoms cause economic losses to farmers. The distribution of *Eimeria* spp. in cattle has, therefore, been reported to have spread widely, especially in the tropics and subtropics. Indonesia is a tropical country at high risk of *Eimeria* infections. This study aimed to identify the prevalence and risk factors related to the levels of eimeriosis in beef cattle originating from different geographic areas in Indonesia.

**Materials and Methods:**

Here, 817 fecal samples were collected from beef cattle in Indonesia, including 282 calves, 535 adults, 530 males, and 287 females. In addition, 156 semi-intensively and 661 intensively managed cattle were randomly collected. Then, fecal samples were analyzed by parasitology examinations.

**Results:**

Screening examination using the sugar flotation modification method showed that Eimeria spp. were prevalent in Indonesia, as 65.4% of the bacterial strain was detected. The prevalence of identified Eimeria spp. in Indonesia was highest in North Maluku (Maluku Island) (94.1%), whereas the lowest levels were observed in West Java (24.0%) (Java Island). The prevalence was also found to be higher in males (79.3%) than females (51.9%). Similarly, levels in semi-intensively managed cattle (66.7%) were higher than those subjected to intensive management (65.9%). However, its prevalence in calf and adult cattle was similar.

**Conclusion:**

Bovine eimeriosis spp. were detected at high prevalence in Indonesia, and high-level risks were observed in infected males, including those under the semi-intensive management. In addition, although the results from oocyst examinations were based on qualitative analysis, the endemicity levels of Eimeria spp. among farms in Indonesia should be considered because Eimeria spp. were distributed in most parts of Indonesia. Based on the results of this study, we provide the first information about the prevalence of bovine eimeriosis from different geographical locations in Indonesia, which have differing climates associated with the level of the existing risk factors. Hence, farmers are advised to pay more attention to strict biosecurity techniques on their farms, thereby favoring the early control of bovine eimeriosis.

## Introduction

Coccidia are protozoan parasites. Consequently, *Eimeria* spp. are among the gastrointestinal protozoans of the genus apicomplexan parasite and subclass coccidia (commonly known to cause coccidiosis) [1]. *Eimeria* spp. are host specific (cattle have their specific infecting *Eimeria* spp. strain). However, Eimeria-infected cows serve as sources of bovine eimeriosis infections [2]. Bovine eimeriosis is difficult to control in livestock because it spread worldwide. *Eimeria* spp. also infect cows orally with an infective stage. The sporulating oocysts that survive in the environment also favor bacterial transmission [3].

Studies have reported that the costs related to morbidity, impaired performance, mortality, and anticoccidial treatment end in substantial economic losses [3,4]. Bovine eimeriosis has, therefore, been estimated to cause economic losses of up to USD 400 million worldwide [2,5]. Lassen and Østergaard [6] also estimated an annual loss of 8-9% due to eimeriosis infections that were simulated in the herd. Thus, further studies established that bovine *Eimeria* infections were prevalent in cattle herds [3,7,8]. Gastrointestinal parasites are recognized as an important disease within the livestock sector, which not only affects health but also affects the productive and reproductive performance of livestock [9]. Moreover, it is assumed that the prices of subclinical diseases outweigh those of clinical eimeriosis [10]. Subclinical cases are, therefore, more common and are proposed to quietly disrupt intestinal physiology, thereby resulting in high feed conversion. However, consistent animal growth does not increase over an extended period compared to clinical cases, which are proposed to be quickly diagnosed and treated [11]. It was also reported that diagnosis based on the qualitative identification of oocysts in feces using the sugar flotation modified method was highly sensitive for detecting *Eimeria* spp. [12]. Indonesia is a tropical country that has a total beef cattle population of approximately 16.5 million, which is more than that of dairy cattle, whose population is only approximately 0.5 million [13]. Nevertheless, cow eimeriosis is a disease that has been neglected in Indonesia. Therefore, it does not get attention from the government and the breeders themselves. Notably, eimeriosis is a parasitic disease that can harm farmers as well by decreasing the performance and productivity of cattle. Thus, beef cattle farms are more sensitive to infections by *Eimeria* spp. [12]. Hence, early prevention through biosecurity and proper diagnosis is a key in determining control and understanding the specific epidemiological conditions in livestock [14].

The standard examination method used in Indonesia as a detection technique for controlling eimeriosis is the flotation technique [12]. However, so far, no national studies have documented the prevalence and distribution of bovine eimeriosis in several major Indonesian provinces. Prevalence can also differ depending on location and farm management systems [2,15]. In addition, Indonesia has different weather conditions in each location, so it is necessary to understand the distribution of *Eimeria* species in these several geographical locations.

This is a preliminary study that determined the prevalence and risk factors related to the level of eimeriosis in beef cattle originating from several different geographic areas in Indonesia using the sugar flotation modification method.

## Materials and Methods

### Ethical approval

The study was approved by Research Ethics Committee, Faculty of Veterinary Medicine, University of Gadjah Mada, Indonesia (Approval no.: 00032/EC-FKH/Int./2020).

### Study period and area

The study was conducted from 4 May to 30 October 2020. Indonesia is that the largest archipelago in the world and there are over 13,466 islands. A sampling method was performed within the following 18 Indonesian main provinces within the major islands: North Sumatera, West Sumatera, Bangka Belitung, West Java, Central Java, Yogyakarta, East Java, Central Sulawesi, Southeast Sulawesi, West Sulawesi, South Sulawesi, Central Kalimantan, East Kalimantan, West Nusa Tenggara Islands, East Nusa Tenggara Islands, North Maluku, Papua, and West Papua.

**Figure-1:**
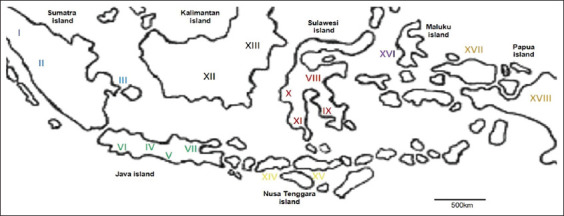
Distribution eimeriosis in cattle by region of cattle herds in Indonesia [Source: https://akupetagambar.blogspot.com/2016/02/peta-indonesia-outline.html].

In this study, 817 fecal samples from 817 beef cattle were obtained. One fecal sample was obtained from each animal. The weather condition during the sample collection period was the dry season. Furthermore, the typical annual temperature on average during this period was 24°C-33°C. In addition, fecal samples were collected as well from 18 provinces, and each examined province had more than 2 farms. The location of those areas is shown in Figure-1. Notably, none of the animals showed clinical symptoms when fecal samples were collected. Moreover, the fecal consistency during sample collection was normal. Fecal samples were taken as well from the rectums of cattle, preserved in plastic bags, and then stored at 4°C until laboratory examinations. Laboratory examinations were conducted at Indonesian Research Center for Veterinary Science and Parasitology Laboratory in Faculty of Veterinary Medicine, Universitas Gadjah Mada.

### Fecal examination

Fecal samples were examined qualitatively using the sugar flotation method as supported by a previous report [12]. Briefly, 1 g of the fecal sample was diluted in 9 mL of water, then centrifuged at 800*× g* for 5 min. Afterward, the supernatant was discarded. Subsequently, 10 mL of sugar solution with a selected gravity of 1.2 (e.g., 100 g of sugar added to 120 mL of distilled water) was added to the sediment. Next, centrifugation was conducted at 800*× g* for 5 min, after which it was placed on a glass slide. The whole smear was examined through microscopy [12,16].

### Statistical analysis

Observed parameters during this study were *Eimeria* spp. prevalence, the distribution of infected cattle, and the high-risk severity of the strain as an indicator of infection. The prevalence was also taken as the number of positive cases divided by the number of tested cases. However, *Eimeria* spp. distribution was compared among location prevalences using analysis of variance at a=5%.

## Results

In our fecal examinations using the sugar flotation modified method to qualitatively identify *Eimeria* spp., all 18 provinces in the major islands of Indonesia were positive for eimeriosis. The positive strains ranged from 24% to 94.12%. Overall, *Eimeria* spp. in Indonesia had a prevalence rate of 65.4%. Furthermore, the prevalence of identified *Eimeria* spp. in Indonesia was highest in the North Maluku province (Maluku Island), with a prevalence of 94.1%; however, the prevalence was lowest in the West Java Province (Java Island) at the rate of 24.0% compared with the other locations (p≤0.05) (Table-1).

**Table 1:** Prevalence of *Eimeria* spp. as per the study areas.

No. of region	Name of province	No. of sample	Positive	%	Average
I	North Sumatera	45	39	86.7	70.3
II	West Sumatera	16	13	81.3	
III	Bangka Belitung	28	12	42.9	
IV	Central Java	60	52	86.7	53.7
V	Yogyakarta	29	12	41.4	
VI	West Java	50	12	24.0	
VII	East Java	78	49	62.8	
VIII	Central Sulawesi	30	22	73.3	68,9
IX	Southeast Sulawesi	60	44	73.3	
X	West Sulawesi	30	21	70.0	
XI	South Sulawesi	49	29	59.2	
XII	Central Kalimantan	51	39	76.5	83
XIII	East Kalimantan	19	17	89.5	
XIV	West Nusa Tenggara	98	49	50.0	58.5
XV	East Nusa Tenggara	70	47	67.1	
XVI	North Maluku	34	32	94.1	94.1
XVII	Papua	49	33	67.4	62.3
XVIII	West Papua	21	12	57.1	
Total		817	534	65.4	

In addition, according to sex and management, the prevalence of bovine eimeriosis was higher in males (79.3%) than females (51.9%). Nevertheless, those subjected to the semi-intensive management were higher (66.7%) than those under intensive care (65.9%). The higher prevalence was also significant (p≤0.05). Likewise, results according to age showed that the prevalence of bovine eimeriosis in calf (69.1%) and adult cattle (69.2%) had a high prevalence of *Eimeria* spp. with no significant difference (p>0.05) (Table-2).

**Table 2:** Prevalence of *Eimeria* spp. as per risk factors.

Risk factor	No. of Sample	Positive	%
Management			
Semi intensive	156	104	66.7
Intensive	661	436	65.9
Sex			
Male	530	420	79.3
Female	287	149	51.9
Age			
Calf (<2 year)	282	195	69.1
Adult (> 2 year)	535	370	69.2

## Discussion

### The sugar flotation method

Although *Eimeria* spp. are not highly pathogenic and do not cause death, these species damage tissues, then increase sensitivity to other contagious infectious diseases. Therefore, the most harmful species of coccidia in ruminants are of the genus Eimeria. In contrast, bovine eimeriosis is an animal infectious disease that causes fatal losses in the form of weight loss, stunted livestock growth, and decreased production. Thus, a management strategy for eimeriosis control in the livestock industry should be considered, especially through detection to find out what *Eimeria* species were breeding on these farms. Strategies toward improper control will increase cases of eimeriosis due to oocysts that continue to contaminate the environment, thereby serving as viable sources of transmission to livestock. Sensitive diagnostic techniques also hold an important role in preventive strategies and in controlling eimeriosis infections in cattle [17].

To enhance these detection processes, the flotation method has been established as a standard parasitological technique. The flotation method is divided into quantitative and qualitative methods. Therefore, in this study, the sample was examined using qualitative methods (sugar centrifugal flotation modification). A qualitative examination of the sample sonly determines the positive and negative presence of *Eimeria* spp. oocysts. However, the modified sugar centrifugal flotation technique is a qualitative method that has been modified to a concentration, which is more sensitive and needs only a few samples to diagnose the presence of *Eimeria* oocysts [12,18].

The Whitlock method is commonly used in Indonesia due to its simplicity, rapidity, and suitability for counting the number of oocysts or eggs in feces in addition to its ease in detecting light or heavy infections [12]. Therefore, the simplest sugar flotation procedure involves mixing a small amount of feces with the sugar solution (specific gravity 1.27) in a centrifuge tube and adding more solutions until the tube is nearly full. The preparation is then allowed to stand until the oocysts float to the top, after which a sample from the top is removed and placed on a microscope slide using a tool, such as a wire loop, straw, needle hub, or a glass rod.

A refinement of this method involves filling the cylinder until a slightly positive meniscus is formed, then placing the glass coverslip over it. Afterward, the tube is allowed to stand until the oocysts have floated to the top, after which the coverslip is removed and placed on a microscope slide for examination. Veterinary hospitals usually use this method based on cost, ease of use, availability of hardware, or simply tradition. However, this method came with high contamination and less sensitivity [19].

A sugar centrifugal flotation modification technique, using a sugar solution of specific gravity 1.27, was evaluated as a sensitive method to successfully recover oocysts from feces (min 10 eggs or oocyst per gram) with low contamination [12,19]. Sensitive examination results are, therefore, important as a method for diagnosing the presence of *Eimeria* oocysts [18]. One oocyst in the intestinal tract reproduces rapidly and causes intestinal damages that impair nutrient absorption and is among the most difficult gastrointestinal parasites to control in cattle farming [3,12]. Bovine eimeriosis can also affect body performance and cattle production, which causes growth delays. These symptoms decrease the quality of livestock, thus resulting in high morbidity and mortality, which can hinder the sustainability of livestock [20]. In addition, eimeriosis is primarily a disease of young animals, but can affect older animals that are in poor condition. Adult cattle were diagnosed with foul smell, bloody diarrhea, anorexia, emaciation condition, smudging of the perineum, and tail with blood-stained dung [21].

### Risk factors for bovine eimeriosis in Indonesia

In this study, a survey of *Eimeria* spp. was conducted in Indonesia. Epidemiological studies based on the frequency of *Eimeria* spp. infection in a different study primarily identified high-risk groups and formulated appropriate interventions. However, we assessed the prevalence rate and associated risk factors.

Research on eimeriosis in Indonesia has been conducted by several researchers, with most of the data obtained coming from the island of Java and only a few from outside the island of Java. A previous examination on the presence of *Eimeria* spp. in beef cattle on traditional farms in West Java by the sugar flotation method in 2014 recorded a prevalence of 22.4% [22]. However, a highly variable prevalence rate of 4.1-59.2% was recorded in 2016 [23], with the results from this study being 24.0%. The prevalence of bovine eimeriosis has also been reported in several areas of Central Java using the flotation method, and the rate in 2013 was 52.7% [24-27], in 2014 was 38.8% [28], in 2016 was 15.33% [15], and in 2019 was 55% [12]. An increase in prevalence was, however, observed in this study 86.7%. This variation in prevalence can also be due to the difference of time (season and temperature) during sampling, examination method used, or other risk factors [18].

In contrast, the overall prevalence of bovine eimeriosis in Indonesia was lower (65.4%) than previously reported (72.07%) [14]. Hamid *et al*. [14] conducted a study to detect the presence of *Eimeria* spp. by parasitology examinations (quantitative method of Mc. Master) in nine provinces of Indonesia from March to October 2017. The previous studies were not specific about the season of sample collection. However, we propose that the time difference accounted for this prevalence variation. Nevertheless, according to the Meteorology, Climatology, and Geophysics Indonesian Agency [29], March is the rainy season in the territory of Indonesia. Therefore, the peak of rainfall should occur in several regions of Indonesia from November to March. The warm and humid tropical climate in Indonesia would, therefore, serve as risk factors for eimeriosis as well since it occurred persistently. Weather and temperature in Indonesia also vary widely. Rainy and dry seasons which intersect these two seasons also exist. Thus, it is reported that the rainy season significantly corresponds to a higher prevalence of bovine eimeriosis [30,31].

The result of this study showed that the highest infection rate was in Maluku island (94.1%), followed by Kalimantan (83%), Sumatra (70.3%), Sulawesi (68.9%), Papua (62.3%), and Nusa Tenggara (58.5%) Islands, however, the lowest infection was in Java Island (53.7%). Therefore, we propose a possible association between risk factors and the prevalence rate detected in different locations. The highest prevalence was also observed on the island of Maluku, which has a tropical climate with an average humidity of 85.4%, and a predominantly semi-intensive system in some rural areas. Eimeriosis also occurs in animals grazed in pastures, especially in the dry season when animals forage around areas that were contaminated with water and soil. Therefore, oocysts were highly resistant in the environment and survived under favorable conditions with suitable humidity and temperature [32].

Variations in the reported prevalence rates can also be attributed to various risk factors, such as the sampling period, sample size, geographic area, and climate as observed in different study areas. It should be borne in mind, however, that the high prevalence of eimeriosis in humid geographic areas explains the reported prevalence from these different locations [32]. Environmental factors that support eimeriosis in cattle, namely, season/climate (temperature and humidity) and livestock management can also influence the prevalence rate of bovine eimeriosis [33,34]. Moreover, eimeriosis occurs more frequently in the winter or rainy season than in the dry season [35,36].

Apart from environmental factors, other risk factors for eimeriosis exist, such as host management, gender, and age, which can influence the prevalence of eimeriosis in a region. *Eimeria* spp. can also infect all types of cattle [37]. However, Oluwadare [38] conducted a study that showed that the prevalence of bovine eimeriosis in Nigeria was higher in beef cattle than in dairy cows. The management of beef maintenance in Indonesia is comprised intensive, semi-intensive, and extensive maintenance systems. Nevertheless, intensive care was mostly used in Indonesia because maintenance is conducted in a cage. However, in several areas in Indonesia, many farmers used a semi-intensive care system, where livestock was cared for through caging and herding. Following this method, cattle are kept in cages from the start until harvest. This type of care is followed in small part rural areas extensively [39].

Management patterns also affect the prevalence of eimeriosis incidents, such as cage density, population density in grazing areas, oxygen and lighting levels, sanitation, drainage, feeding systems, and drinking sources [40,41]. Prevalence of infection and intensity of *Eimeria* spp. in cattle are also lower in pen than pasture [42]. Thus, cows shed a lot of oocysts through feces in their closed pens every day during the patent period, which can increase parasite transmission and increase the reproductive rate of *Eimeria* spp. The most common symptoms of eimeriosis appear 2-3 weeks after infection in a newly contaminated environment [6].

Regarding the previousstudy [43], supporting management systems have recorded that a high prevalence of eimeriosis was higher in intensive systems, which can be caused by food contaminated by oocysts derived from feces that accumulate around the intensive cage. Analysis of extrinsic associated risk factors also revealed the floor type, feeding, and watering systems, in addition to the herd size as significant risk factors for *Eimeria* spp. infections [32]. Another finding that prompted this study was that the prevalence of bovine eimeriosis was higher (66.7%) in the semi-intensive system compared to the intensive system (65.9%). Intensive system maintenance is used in beef cattle in Indonesia because it is more efficient in terms of feeding, cage cleaning, disease management, and bathing livestock. However, in marginal areas that still use semi-intensive systems, such as the North Maluku and Kalimantan areas, cattle rearing is based on integration with crops, such as oil palm. In the intensive system, cattle are housed for 24 h, whereas in the semi-intensive system, the opposite is the case, where cattle can move to graze the field. Thus, the chance for contamination with *Eimeria* oocysts through the grass is reduced. Nevertheless, although the cattle in an intensive system results in increased prevalence, if sanitation is not maintained properly, it will lead to a high prevalence of bovine parasitic diseases [43,44].

Studies have also shown that the intensity of infections by *Eimeria* spp. in males (79.3%) was higher than in females (51.9%). The same results were reported by Kertawirawan *et al*. [43]. Another study reported that the prevalence of eimeriosis was higher in males than in females because males were rarely cleaned compared to female that was always cleaned at the time of milking [18]. Therefore, the higher prevalence of males was proposed to have something to do with the frequency of herding. Therefore, since males were grazed more frequently and explored more areas than female, the chance of being infected with *Eimeria* spp. was increased. A level of contamination has also been suspected in the grazing field, and the possibility of *Eimeria* transmission occurs when pasturing in areas that have been contaminated by *Eimeria* [23]. From our results, the prevalence of eimeriosis was based on age, with no significant difference (p>0.05). Nevertheless, the prevalence in adults (>2 year) was higher than in calves (<2 years). Bovine eimeriosis is a disease that attacks young animals. Thus, infected cattle were 3 weeks-6 months [43]. The ideal Indonesian beef cattle age is between 1.5 and 2.5 years. Hence, the dominant age of beef cattle samples obtained from farms was around 1.5 to 2.5 years.

Our results also propose that *Eimeria* spp. was more widespread in Indonesia. Therefore, it needed early control and attention from the Indonesian government to maintain the performance of cattle farming in Indonesia. Thus, based on the risk factors, bovine eimeriosis occurs ubiquitously in Indonesia and is proposed to specially influence the subclinical presentations of the infection by affecting production parameters in conventional cattle management systems. Therefore, *Eimeria* cases can be reduced if farmers take strict biosecurity measures and good management practices, such as maintaining hygiene, including sanitation and management of feeding in semi-intensive systems. Furthermore, as many species exist that can infect cattle, it is imperative to identify which of them has more occurrence. Nevertheless, integrated strategies should be used to prevent and control *Eimeria* spp. infections. Likewise, to control the spread of the disease, a proper sensitive diagnosis is needed to determine how to deal with these infections [45].

## Conclusion

Based on our results, the prevalence of *Eimeria* spp. in beef cattle in Indonesia was high. This study showed a high rate of *Eimeria* spp. infections in traditional farms (semi-intensive management) in North Maluku. These results also indicated that *Eimeria* spp. spread in most parts of Indonesia. Therefore, a need exists to establish efficient control measures that improve animal health and performance by lowering the gastrointestinal parasitism of cattle. In addition, further epidemiological investigations on *Eimeria* spp. are also needed to investigate the effect of other risk factors, such as breed, watering system, and herd size on infection rates.

## Authors’ Contributions

FE, RWN, LFW, and DHS: Collected samples. FE, RWN, and LFW: Analyzed the samples. FE, RWN, AHW, JP, and DP: Wrote original draft and revised the manuscript. All authors read and approved the final manuscript.

## References

[1] Lucas A.S, Swecker W.S, Lindsay D.S, Scaglia G, Neel J.P.S, Elvinger F.C, Zajac A.M (2014). A study of the level and dynamics of *Eimeria* populations in naturally infected, grazing beef cattle at various stages of production in the Mid-Atlantic USA. Vet. Parasitol.

[2] Matjila P.T, Penzhorn B.L (2002). Occurrence and diversity of bovine coccidia at three localities in South Africa. Vet. Parasitol.

[3] Daugschies A, Najdrowski M (2005). Eimeriosis in cattle:Current understanding. J. Vet. Med. B Infect. Dis. Vet. Public Health.

[4] Hermosilla C, Zahner H, Taubert A (2006). *Eimeria bovis* modulates adhesion molecule gene transcription in and PMN adhesion to infected bovine endothelial cells. Int. J. Parasitol.

[5] Bruhn F.R.P, Lopes M.A, Demeu F.A, Perazza C.A, Pedrosa M.F, Guimarães A.M (2011). Frequency of species of *Eimeria* in females of the Holstein-Friesian breed at the postweaning stage during autumn and winter. Rev. Bras. Parasitol. Vet.

[6] Lassen B, Østergaard S (2012). Estimation of the economical effects of *Eimeria* infections in Estonian dairy herds using a stochastic model. Prev. Vet. Med..

[7] Autzen S, Maddox-Hyttel C, Virge H, Monrad J (2002). Infection with *Eimeri*a species in calves:Assessment of risk factors and correlations between diarrhea and oocyst secretion. Dansk Vet. Tidskr.

[8] Indraswari A.A.S, Suwiti N.K, Apsari I.A.P (2017). *Eimeria auburnensis* and *Eimeria bovis* of protozoa gastrointestinal infected on female Bali Cattle in Nusa Penida. J. Vet. Udayana.

[9] Alemnew E, Delil F, Addis H (2017). Prevalence of bovine coccidiosis and ostertagiosis in and around Kombolcha district of south Wollo, Ethiopia. Academ Arena.

[10] Faber J.E, Kollmann D, Heise A, Bauer C, Failing K, Bürger H.J, Zahner H (2002). *Eimeria* infections in cows in the periparturient phase and their calves:Oocyst excretion and levels of specific serum and colostrum antibodies. Vet. Parasitol.

[11] Forslid A, Christensson D, Dahl J, Grandi G, Enemark J (2015). Bovine eimeriosis in Swedish calves:epidemiology and insights into sampling procedures. Vet. Parasitol. Reg. Stud. Reports.

[12] Ekawasti F, Nurcahyo W, Wardhana A.H, Shibahara T, Tokoro M, Sasai K, Matsubayashi M (2019). Molecular characterization of highly pathogenic Eimeria species among beef cattle on Java Island, Indonesia. Parasitol. Int.

[13] MoARI (2018). Livestock and Animal Health Statistic.

[14] Hamid P.H, Kristianingrum Y.P, Prastowo S (2019). Bovine coccidiosis cases of beef and dairy cattle in Indonesia. Vet. Parasitol. Reg. Stud. Reports.

[15] Hamid P.H, Kristianingrum Y.P, Prastowo J, da Silva L.M.R (2016). Gastrointestinal parasites of cattle in Central Java. Am. J. Anim. Vet. Sci.

[16] Matsubayashi M, Takami K, Kimata I, Nakanishi T, Tani H, Sasai K, Baba E (2005). Survey of *Cryptosporidium* spp. and *Giardia* spp. Infections in various animals at a zoo in Japan. J. Zoo Wildl. Med.

[17] Heidari H, Gharekhani J (2014). Detection of *Eimeria* species in Iranian Native Cattle. Int. J. Adv. Res.

[18] Ekawasti F, Wardhana A.H (2019). Coccidiosis disease in cattle in Indonesia and development of diagnostic techniques. J. Wartazoa.

[19] Matsuo K, Kamiya H (2005). Modified sugar centrifugal flotation technique for recovering *Echinococcus multilocularis* eggs from soil. J. Parasitol.

[20] Marquez J.C (2014). Calf Intestinal Health:Assessment and Diatery Interventions for its Improvement.

[21] Sudhakara R.B, Sivajothi S, Rayulu V.C (2015). Clinical coccidiosis in adult cattle. J. Parasit. Dis.

[22] Ananta S.M, Suharno, Hidayat A, Matsubayashi M (2014). Survey on gastrointestinal parasites and detection of *Cryptosporidium* spp. on cattle in West Java, Indonesia. Asian Pac. J. Trop. Med.

[23] Sufi I, Sudarnika E (2016). Prevalence and risk factor of Coccidiosis in dairy cattle in Bandung district. Indonesian J. Vet. Sci.

[24] Nugroho W.S (2013). Prevalence of Coccidiosis in Calves in Wonogiri Regency Thesis.

[25] Sumiarto B (2013). Prevalence and risk factors for coccidiosis (*Eimeria* spp.) In calves in Boyolali district Thesis.

[26] Budiharta S (2013). Prevalence of Coccidiosis in Calves in Klaten Regency, Central Java Thesis.

[27] Raharjo (2013). Incidence Rate of Coccidiosis in Dairy Cattle Calves in the Wetan Wukirsari Cangkringan, Sleman Livestock Group Thesis.

[28] Nanditya (2014). Prevalence of Coccidiosis in Cows and Prevalence of Calf Mortality in Sragen, Central Java, Indonesia:A Case Study Thesis.

[29] BMKG (2020). The Meteorology, Climatology and Geophysics.

[30] Manya P, Sinha S.R.P, Sinha S, Verma S.B, Sharma S.K, Mandal K.G (2008). Prevalence of bovine coccidiosis at Patna. J. Vet. Parasitol.

[31] Gupta A, Singh N.K, Singh H, Rath S.S (2016). Assessment of risk factors associated with prevalence of coccidiosis in dairy animals of Punjab. J. Parasit. Dis.

[32] Lopez-Osorio S, Villar D, Failing K, Taubert A, Hermosilla C, Chaparro-Gutierrez J.J (2020). Epidemiological survey and risk factor analysis on *Eimeria* infections in calves and young cattle up to 1 year old in Colombia. Parasitol. Res.

[33] Abebe R, Kumesa B, Wessene A (2008). Epidemiology of *Eimeria* infections in calves in Addis Ababa and Debre Zeit Dairy Farms, Ethiopia Intern. J. Appl. Res. Vet. Med.

[34] Matsubayashi M, Kita T, Narushima T, Kimata I, Tani H, Sasai K, Baba E (2009). Coprological survey of parasitic in pigs and cattle in slaughterhouses in Osaka, Japan. J. Vet. Med. Sci.

[35] Pfukenyi D.M, Mukaratirwa S, Willingham A.L, Monrad J (2007). Epidemiological studies of parasitic gastrointestinal nematodes, cestodes and *Coccidia* infections in cattle in the Highveld and Lowveld Communal Grazing Areas of Zimbabwe. Onderstepoort J. Vet. Res.

[36] Makau D.N (2014). A study of factors associated with the prevalence of *Coccidia* infection in cattle and its spatial epidemiology in Busia, Bungoma and Siaya Counties, Kenya Thesis.

[37] Makau D.N, Gitau G.K, Muchemi G.K, Thomas L.F, Cook E.A, Wardrop N.A, Fevre E.M, de Glanville W.A (2017). Environmental predictors of bovine *Eimeria* infection in western Kenya. Trop. Anim. Health Prod.

[38] Oluwadare A.T (2004). Studies on Bovine *Coccidia* [Apicomplexia:Eimeriidae] in Parts of Plateau State, Nigeria Thesis.

[39] Qoimah H.R (2017). Feeding Management and Adequacy Evaluation Nutrients in Ongole Bunting Cows in PT. Karya Anugerah Rumpin Desa Cibodas, Kecamatan Rumpin, Bogor District, West Java Thesis.

[40] Rehman T.U, Khan M.N, Sajid M.S, Abbas R.Z, Arshad M, Iqbal Z, Iqbal A (2011). Epidemiology of *Eimeria* and associated risk factor in cattle of district Toba Tek Singh, Pakistan. Parasitol. Res.

[41] Bangoura B, Daugschies A, Fuerll M (2007). Influence of experimental *Eimeria zuernii* infection on clinical blood chemistry in calves. Vet. Parasitol.

[42] Jäger M, Gualy M, Bauer C, Failing K, Erhardt G, Zahner H (2005). Endoparasites in calves of beef cattle herds:Management systems dependent and genetic influences. Vet. Parasitol.

[43] Kertawirawan I.P.A, Budiari L.G, Sutresna I.N (2019). Identification of *Eimeria* sp. Prevalence in Bali Cattle on Marginal Land with Semi Intensive Cultivation Patterns. Proceedings of the National Seminar on Agricultural Resource Readiness and Site-Specific Innovation Entering the Industrial Age 4.0.

[44] Volkandari S.D, Sudrajad P, Prasetyo D, Subiharta, Prasetyo A, Pujianto J, Cahyadi M (2019). Impact of Intensive and Semi-Intensive Maintenance Systems on Bali Cattle Body Size at Bali Animal Breeding Center (BPTU) Bali Cattle. Proceedings of the National Seminar on Agricultural Resource Readiness and Site-Specific Innovation Entering the Industrial Age 4.0.

[45] Hussin A.G (2016). Prevalence and associated risk factors of *Eimeria* spp. in Cattle of Baghdad, Iraq. J. Appl. Anim. Sci.

